# Not out-of-reach: Engaging the older old isolated African Americans with and without MCI

**DOI:** 10.1093/geroni/igab046.2392

**Published:** 2021-12-17

**Authors:** Laura Struble, Kathleen Potempa, Benjamin Hampstead, Alexis Ellis, Jesica Pedroza, Peter Lichtenberg, Hiroko Dodge

**Affiliations:** 1 University of Michigan School of Nursing, Ann Arbor, Michigan, United States; 2 University of Michigan, University of Michigan, Ann Arbor, Michigan, United States; 3 University of Michigan, Ann Arbor, Michigan, United States; 4 University of Michigan, Ypsilanti, Michigan, United States; 5 University of Michigan, University of Michigan, Michigan, United States; 6 Wayne State University, Detroit, Michigan, United States; 7 Oregon Health & Science University, Portland, Oregon, United States

## Abstract

The Internet-Based Conversational Engagement Clinical Trial (I-CONECT, ClinicalTrials.gov: NCT02871921) is a multi-center randomized, 12-month efficacy study. There is converging evidence that social isolation is a risk factor of cognitive decline and dementia. We hypothesized that increasing social interaction in older adults with normal cognition or mild cognitive impairment (MCI) could improve or sustain cognitive function through internet-based conversational engagement. African Americans (AA) are at higher risk for developing dementia but their participation in clinical trials is low. Objectives: (1) discuss the effective outreach process to recruit urban AA older old adults (mean targeted age of 80+); (2) describe how we retained participants in a yearlong study using technology-based interventions. The most successful outreach and recruitment sources were the voter registration mass mailings and the Healthier Black Elders Research Center. Successful recruitment methods included: hiring diverse staff, compensating participants’ time, and adjusting research protocols for opting out of MRIs and genetic saliva samples. Technology intervention strategies included: providing user-friendly Chromebooks and free internet connections, simple instructions with pictures, vision and hearing correction, and in-home training with technology support backup. During the pandemic, we could assists participants in learning to use the laptop remotely. Over 12,000 subjects were contacted, which led to 39 randomized participants. Our retention rate thus far is over 75%. This demonstrates that AA older adults are reachable, willing to participate in research and able to use communication technology with appropriate supports for long-term sustainable interaction that may improve cognition and health equity.

